# Construction of the reaction mechanism for O_3_ oxidation desulfurization and denitrification based on the minimized reaction network method

**DOI:** 10.1039/d6ra05271a

**Published:** 2026-10-01

**Authors:** Hongjing Yu, Wenwen Xia, Li Yao

**Affiliations:** a Jiangsu Maritime Institute Nanjing 211170 China hongjingyu@jmi.edu.cn; b Dalian Maritime University Dalian 116026 China; c Merchant Marine College, Shanghai Maritime University Shanghai 201306 China yaoli@shmtu.edu.cn

## Abstract

The applicable O_3_ oxidation mechanism for simultaneous desulfurization and denitrification is of great significance in the research on integrated removal technology for ship exhaust pollutants. Based on the minimized reaction network (MRN) method, the reaction mechanism for ozone (O_3_) oxidation desulfurization and denitrification is established in this study. First, a minimal reaction mechanism for O_3_ self-decomposition involving 5 species and 5 reactions is established. Then, based on this O_3_ self-decomposition reaction mechanism, 17 nitrogen-containing species and 26 nitrogen-related reactions are added to form the reaction mechanism for O_3_ oxidation denitrification. Finally, 2 sulfur-containing species and 2 sulfur-related reactions are added to the O_3_ oxidation denitrification reaction mechanism to construct the complete minimal reaction mechanism for O_3_ oxidation desulfurization and denitrification. Therefore, the reaction mechanism includes 24 species and 33 reactions. The reliability and practicality of the reaction mechanism are verified by comparing the numerical simulation results under different operating conditions with findings from different researchers.

## Abstract 2

Based on the minimized reaction network method, a reaction mechanism for O_3_ oxidation desulfurization and denitrification was constructed. The mechanism shows good prediction performance, and it is more suitable for engineering calculations.

## Graphical abstract

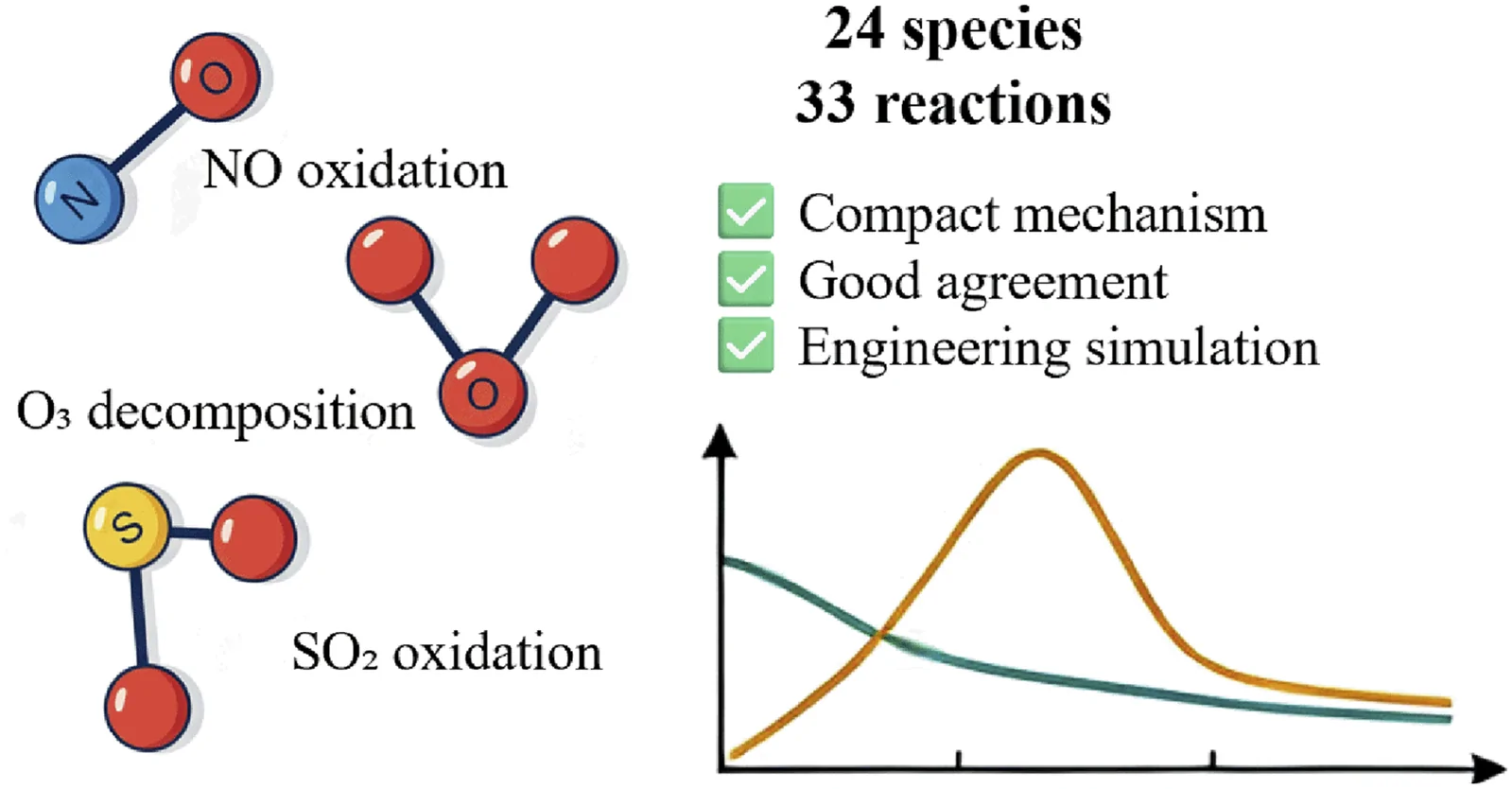

## 1. Introduction

Owing to its economic advantages such as low operational costs and large cargo-carrying capacity, maritime transport accounts for more than 80% of global freight volume. Transport structure restructuring and the year-on-year growth of freight turnover jointly promote the sustained expansion of the shipping industry. At present, the application of clean fuels in vessels still has many limitations. In addition, retrofitting existing vessels involves great difficulties. In the foreseeable future, ocean-going vessels will still rely on traditional diesel engines as their primary propulsion equipment. Data indicate that the annual emissions of sulfur oxides (SO_*x*_) and nitrogen oxides (NO_*x*_) from global ships reach 15 million tons and 25 million tons, respectively, severely endangering global environmental quality and public health.^1–3^ With the continuous expansion of global trade, the number of vessels increases year by year and shipping routes become increasingly dense. Consequently, the adverse impacts of ship exhaust emissions on the atmospheric environment and human health are becoming increasingly severe. Accordingly, it is imperative to control ship exhaust pollutant emissions. In addition, considering the economic requirements and space constraints of vessels, low-cost integrated emission control technologies for multiple pollutants with a small footprint conform to the development trend of ship exhaust pollution prevention and control. According to the related researches, the O_3_ oxidation–absorption method can achieve integrated and efficient removal of multiple pollutants. This technology presents high application value and broad development prospects in the field of integrated pollutant removal from ship exhaust.^4–12^

Numerical simulation based on reaction mechanisms can predict pollutant removal performance under different working conditions and achieve precise control for the integrated removal of ship exhaust pollutants. At present, there is no complete and available mechanism for O_3_ oxidation desulfurization and denitrification in existing studies. Moreover, the limited O_3_ oxidation denitrification mechanisms fail to consider the accuracy of the O_3_ self-decomposition sub-mechanism. Besides, the existing reaction mechanisms developed by different researchers present discrepancies in reaction networks and species.^6,12–15^ Therefore, constructing a complete and applicable O_3_ oxidation desulfurization and denitrification reaction mechanism is of great significance to the research on integrated ship exhaust pollutant removal technologies.

## 2. Construction of the reaction mechanism

### 2.1 Minimized reaction network (MRN) method

All complex chemical reactions eventually attain equilibrium. Accordingly, the concentrations of the reactants and products upon reaction completion depend on the equilibrium constant. In a complex equilibrium system, given the number of chemical species (*N*) and the rank of the atomic matrix (*L*), the number of independent reactions (*F*) is finite, as shown in eqn (1).^16^

1 *F* = *N* − *L*

### 2.2 Construction of the reaction mechanism

First, the number of species was determined. The number of species and reactions for existing O_3_ oxidation denitrification mechanisms is compared in Table 1. Therefore, the proposed O_3_ oxidation denitrification mechanism includes a more comprehensive set of reactive species and considers O_3_ self-decomposition. Moreover, two species for desulfurization are supplemented. Therefore, the reaction mechanism for O_3_ oxidation desulfurization and denitrification established in this study retains 24 species (NO, O_3_, H_2_, SO_2_, O_2_, O˙, NO_2_, 
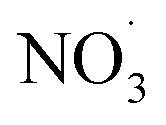
, N_2_O, N_2_O_3_, N_2_O_4_, N_2_O_5_, N_2_, N˙, H_2_O, H_2_O_2_, ˙OH, 
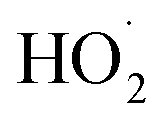
, H˙, HNO_3_, HNO_2_, SO_3_, Ar, and He) adopted from existing mechanisms, as listed in Table 2.^6,12–15^ Among them, the thermodynamic data of SO_2_ and SO_3_ were obtained from RMG and those of the others were obtained from previous research results.^17,18^

**Table 1:** Number of species and reactions in the reaction mechanism for denitrification by O_3_ oxidation

Mechanism	Number of species	Number of reactions
2006-Mok^6^	7	12
2006-Wang^12^	20	65
2014-Asif^13^	9	15
2015-Wang^14^	20	76
2016-Lin^15^	12	24
This work	22	31

**Table 2:** Thermodynamic data of the species

Species	Temperature	*a*_1_	*a*_2_	*a*_3_	*a*_4_	*a*_5_	*a*_6_	*a*_7_
O_3_	Below 1000 K	2.73	6.70 × 10^−3^	−4.32 × 10^−7^	−5.35 × 10^−9^	2.83 × 10^−12^	2.28 × 10^4^	11.81
	Above 1000 K	4.97	2.48 × 10^−3^	−1.22 × 10^−6^	2.72 × 10^−10^	−2.28 × 10^−14^	2.21 × 10^4^	−0.21
O_2_	Below 1000 K	3.82	−3.20 × 10^−3^	9.87 × 10^−6^	−9.49 × 10^−9^	3.13 × 10^−12^	−1.07 × 10^3^	3.46
	Above 1000 K	3.24	1.47 × 10^−3^	−7.00 × 10^−7^	1.53 × 10^−10^	−1.26 × 10^−14^	−1.06 × 10^3^	5.64
O˙	Below 1000 K	2.50	−7.33 × 10^−17^	1.76 × 10^−19^	−1.75 × 10^−22^	6.14 × 10^−26^	2.90 × 10^4^	4.08
	Above 1000 K	2.50	1.49 × 10^−17^	−1.10 × 10^−20^	3.37 × 10^−24^	−3.67 × 10^−28^	2.90 × 10^4^	4.08
NO	Below 1000 K	3.87	−2.95 × 10^−3^	7.48 × 10^−6^	−6.11 × 10^−9^	1.74 × 10^−12^	9.43 × 10^3^	3.20
	Above 1000 K	2.96	1.70 × 10^−3^	−7.86 × 10^−7^	1.67 × 10^−10^	−1.35 × 10^−14^	9.56 × 10^3^	7.32
NO_2_	Below 1000 K	3.60	1.04 × 10^−3^	8.87 × 10^−6^	−1.16 × 10^−8^	4.35 × 10^−12^	2.22 × 10^3^	7.71
	Above 1000 K	4.28	3.22 × 10^−3^	−1.55 × 10^−6^	3.42 × 10^−10^	−2.83 × 10^−14^	1.83 × 10^3^	3.19
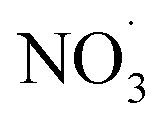	Below 1000 K	2.67	1.65 × 10^−2^	−1.40 × 10^−5^	3.69 × 10^−9^	4.63 × 10^−13^	7.03 × 10^3^	13.71
	Above 1000 K	7.28	3.34 × 10^−3^	−1.65 × 10^−6^	3.70 × 10^−10^	−3.10 × 10^−14^	5.81 × 10^3^	−9.95
N_2_O	Below 1000 K	2.41	1.05 × 10^−2^	−1.23 × 10^−5^	8.39 × 10^−9^	−2.46 × 10^−12^	8.06 × 10^3^	10.06
	Above 1000 K	4.36	3.50 × 10^−3^	−1.62 × 10^−6^	3.47 × 10^−10^	−2.82 × 10^−14^	7.59 × 10^3^	0.32
N_2_O_3_	Below 1000 K	5.60	1.53 × 10^−2^	−1.58 × 10^−5^	9.02 × 10^−9^	−2.28 × 10^−12^	1.14 × 10^4^	2.61
	Above 1000 K	8.98	4.68 × 10^−3^	−2.24 × 10^−6^	4.91 × 10^−10^	−4.05 × 10^−14^	1.05 × 10^4^	−14.63
N_2_O_4_	Below 1000 K	4.19	2.60 × 10^−2^	−2.68 × 10^−5^	1.40 × 10^−8^	−3.03 × 10^−12^	5.91 × 10^3^	8.60
	Above 1000 K	10.44	6.59 × 10^−3^	−3.19 × 10^−6^	7.03 × 10^−10^	−5.83 × 10^−14^	4.31 × 10^3^	−23.16
N_2_O_5_	Below 1000 K	2.75	3.79 × 10^−2^	−4.14 × 10^−5^	2.30 × 10^−8^	−5.29 × 10^−12^	1.61 × 10^3^	16.03
	Above 1000 K	11.85	8.53 × 10^−3^	−4.14 × 10^−6^	9.15 × 10^−10^	−7.60 × 10^−14^	−7.02 × 10^2^	−29.99
N_2_	Below 1000 K	3.66	−1.08 × 10^−3^	1.87 × 10^−6^	−2.36 × 10^−10^	−3.35 × 10^−13^	−1.06 × 10^3^	2.36
	Above 1000 K	2.74	1.77 × 10^−3^	−7.59 × 10^−7^	1.53 × 10^−10^	−1.18 × 10^−14^	−8.31 × 10^2^	7.04
N˙	Below 1000 K	2.50	−7.33 × 10^−17^	1.76 × 10^−19^	−1.75 × 10^−22^	6.14 × 10^−26^	5.52 × 10^4^	4.17
	Above 1000 K	2.50	1.49 × 10^−17^	−1.10 × 10^−20^	3.37 × 10^−24^	−3.67 × 10^−28^	5.52 × 10^4^	4.17
H_2_O	Below 1000 K	3.84	5.36 × 10^−4^	−2.46 × 10^−7^	1.95 × 10^−9^	−1.19 × 10^−12^	−2.82 × 10^4^	0.64
	Above 1000 K	2.80	2.73 × 10^−3^	−7.14 × 10^−7^	7.74 × 10^−11^	−2.16 × 10^−15^	−2.79 × 10^4^	6.20
H_2_O_2_	Below 1000 K	3.22	2.52 × 10^−3^	7.08 × 10^−6^	−1.06 × 10^−8^	4.32 × 10^−12^	−1.43 × 10^4^	9.22
	Above 1000 K	3.42	4.49 × 10^−3^	−1.60 × 10^−6^	2.77 × 10^−10^	−1.89 × 10^−14^	−1.45 × 10^4^	7.65
˙OH	Below 1000 K	3.43	5.96 × 10^−4^	−1.81 × 10^−6^	2.20 × 10^−9^	−7.75 × 10^−13^	3.43 × 10^3^	1.76
	Above 1000 K	2.94	8.09 × 10^−4^	−1.09 × 10^−7^	−1.39 × 10^−11^	3.20 × 10^−15^	3.64 × 10^3^	4.59
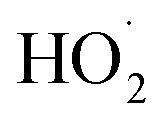	Below 1000 K	3.74	−6.12 × 10^−4^	9.91 × 10^−6^	−1.18 × 10^−8^	4.42 × 10^−12^	8.34 × 10^2^	6.04
	Above 1000 K	3.57	3.13 × 10^−3^	−1.26 × 10^−6^	2.42 × 10^−10^	−1.81 × 10^−14^	7.43 × 10^2^	6.19
H˙	Below 1000 K	2.50	−7.33 × 10^−17^	1.76 × 10^−19^	−1.75 × 10^−22^	6.14 × 10^−26^	2.55 × 10^4^	−0.47
	Above 1000 K	2.50	1.49 × 10^−17^	−1.10 × 10^−20^	3.37 × 10^−24^	−3.67 × 10^−28^	2.55 × 10^4^	−0.47
H_2_	Below 1000 K	3.46	2.67 × 10^−4^	−7.21 × 10^−7^	7.38 × 10^−10^	−1.79 × 10^−13^	−1.04 × 10^3^	−4.13
	Above 1000 K	3.28	9.07 × 10^−5^	2.91 × 10^−7^	−1.06 × 10^−10^	1.08 × 10^−14^	−9.40 × 10^2^	−2.94
HNO_3_	Below 1000 K	0.96	2.46 × 10^−2^	−2.45 × 10^−5^	1.19 × 10^−8^	−2.16 × 10^−12^	−1.52 × 10^4^	20.20
	Above 1000 K	6.60	6.62 × 10^−3^	−2.94 × 10^−6^	6.13 × 10^−10^	−4.90 × 10^−14^	−1.66 × 10^4^	−8.29
HNO_2_	Below 1000 K	3.32	6.70 × 10^−4^	1.79 × 10^−5^	−2.21 × 10^−8^	8.16 × 10^−12^	−5.66 × 10^3^	9.61
	Above 1000 K	3.68	6.80 × 10^−3^	−3.09 × 10^−6^	6.52 × 10^−10^	−5.24 × 10^−14^	−6.11 × 10^3^	5.98
SO_2_	Below 1000 K	3.57	7.84 × 10^−3^	−4.42 × 10^−6^	−2.21 × 10^−9^	2.00 × 10^−12^	4.60 × 10^4^	9.97
	Above 1000 K	6.32	6.52 × 10^−4^	−2.25 × 10^−7^	3.29 × 10^−11^	−2.17 × 10^−15^	4.53 × 10^4^	−4.18
SO_3_	Below 1000 K	2.76	1.34 × 10^−2^	−7.34 × 10^−6^	−1.55 × 10^−9^	1.80 × 10^−12^	−4.90 × 10^4^	11.46
	Above 1000 K	6.70	3.83 × 10^−3^	−1.74 × 10^−6^	3.50 × 10^−10^	−2.60 × 10^−14^	−5.01 × 10^4^	−9.12
Ar	Below 1000 K	2.50	−7.33 × 10^−17^	1.76 × 10^−19^	−1.75 × 10^−22^	6.14 × 10^−26^	−7.44 × 10^2^	4.36
	Above 1000 K	2.50	1.49 × 10^−17^	−1.10 × 10^−20^	3.37 × 10^−24^	−3.67 × 10^−28^	−7.40 × 10^2^	4.35
He	Below 1000 K	2.50	−7.33 × 10^−17^	1.76 × 10^−19^	−1.75 × 10^−22^	6.14 × 10^−26^	−7.44 × 10^2^	0.91
	Above 1000 K	2.50	1.49 × 10^−17^	−1.10 × 10^−20^	3.37 × 10^−24^	−3.67 × 10^−28^	−7.40 × 10^2^	0.91

Then, the number of independent chemical reactions was determined. 22 Reactive species consist of only four elements, namely, N, O, H and S. Accordingly, NO, O_3_, H_2_ and SO_2_, containing the above-mentioned four elements, are selected as the initial species. The remaining 18 species can be generated from these four species. The corresponding matrix of atomic coefficients is presented in Table 3. Based on this matrix, the equations for the independent reactions can be derived and are listed in Table 4.

**Table 3:** Matrix of atomic coefficients

Label	Species	O_3_	NO	H_2_	SO_2_
1	O_2_	2/3	0	0	0
2	O˙	1/3	0	0	0
3	NO_2_	1/3	1	0	0
4	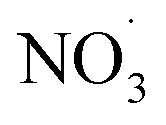	2/3	1	0	0
5	N_2_O	−1/3	2	0	0
6	N_2_O_3_	1/3	2	0	0
7	N_2_O_4_	2/3	2	0	0
8	N_2_O_5_	1/3	2	0	0
9	N_2_	−2/3	2	0	0
10	N˙	−1/3	1	0	0
11	H_2_O	1/3	0	1	0
12	H_2_O_2_	2/3	0	1	0
13	˙OH	1/3	0	1/2	0
14	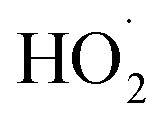	2/3	0	1/2	0
15	H˙	0	0	1/2	0
16	HNO_3_	2/3	1	1/2	0
17	HNO_2_	1/3	1	1/2	0
18	SO_3_	1/3	0	0	1

**Table 4:** Equations for independent chemical reactions

Label	Chemical equation	Label	Chemical equation
1	3O_2_ = 2O_3_	10	3N˙ = 3NO − O_3_
2	3O˙ = O_3_	11	3H_2_O = O_3_ + 3H_2_
3	3NO_2_ = 3NO + O_3_	12	3H_2_O_2_ = 2O_3_ + 3H_2_
4	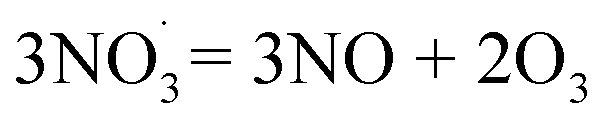	13	6˙OH = 2O_3_ + 3H_2_
5	3N_2_O = 6NO − O_3_	14	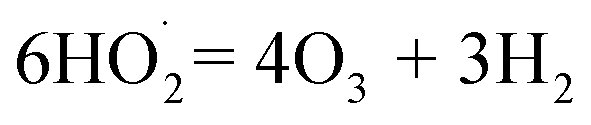
6	3N_2_O_3_ = 6NO + O_3_	15	2H˙ = H_2_
7	3N_2_O_4_ = 6NO + 2O_3_	16	6HNO_3_ = 6NO + 4O_3_ + 3H_2_
8	3N_2_O_5_ = 6NO + O_3_	17	6HNO_2_ = 6NO + 2O_3_ + 3H_2_
9	3N_2_ = 6NO − 2O_3_	18	3SO_3_ = 3SO_2_ + O_3_

It should be noted that the independent reactions are stoichiometric equations satisfying chemical equilibrium conditions, which are not necessarily elementary reactions. Therefore, non-elementary reactions need to be reasonably broken down and replaced for the kinetic data.

Based on previous research results, the diagram for the conversion reaction paths of NO_*x*_ in the O_3_ oxidation denitrification process is given in Fig. 1.^18–20^ This diagram mainly presents the major reaction pathways with energy barriers lower than 120 kJ mol^−1^ during oxidative denitrification. According to the figure, the independent reactions of the O_3_ oxidation denitrification mechanism were split and substituted. Major reactions in the O_3_ oxidation denitrification mechanism were retained. Meanwhile, both the accuracy and conciseness of the mechanism model were guaranteed.

**Fig. 1:**
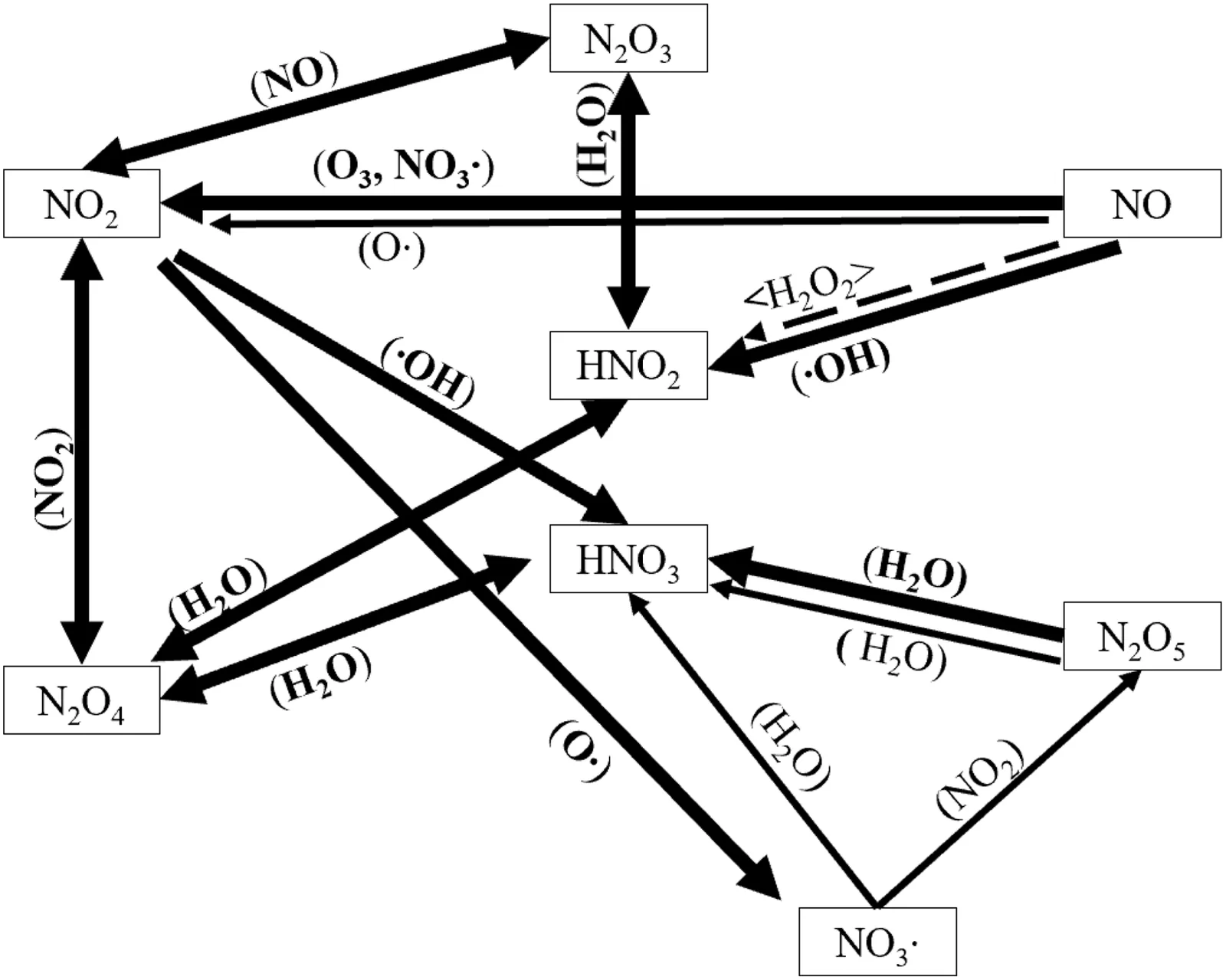
Diagram for the conversion reaction paths of NO_*x*_ in the process of O_3_ oxidation denitrification.

The reaction network (33 reactions) and kinetic data of the final constructed reaction mechanism are summarized in Table 5. The kinetic parameters were obtained *via* two-parameter fitting of the data.^6,12–15,18,20–33^

**Table 5:** Reaction network and kinetic data

Label	Reaction	*A* (cm^3^ mol^−1^ s^−1^)	*E* (kJ mol^−1^)	Ref.
1	O_3_ → O_2_ + O˙	1.31 × 10^9^	22.26	6 and 12–15
2	O_3_ + O_3_ → O_2_ + O_2_ + O_2_	1.81 × 10^10^	10.00	21
3	O_3_ + O_2_ → O_2_ + O_2_ + O˙	1.90 × 10^10^	11.00	22–24
4	O_3_ + O˙ → 2O_2_	4.82 × 10^12^	4.09	15
5	2O˙ → O_2_	1.20 × 10^17^	0.00	12 and 25
6	NO + O_3_ → NO_2_ + O_2_	2.98 × 10^10^	3.00	6 and 12–15
7	NO + O˙ → NO_2_	1.53 × 10^12^	5.53	6 and 12–15
8	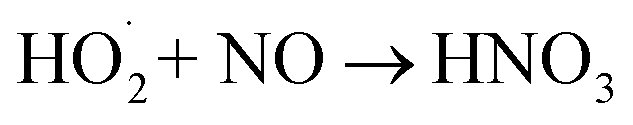	9.19 × 10^11^	154.02	14
9	NO + ˙OH → HNO_2_	5.45 × 10^17^	0.00	12 and 14
10	H_2_O_2_ + NO → HNO_2_ + ˙OH	4.47 × 10^10^	17.54	20
11	NO + NO_3_ → 2NO_2_	1.08 × 10^12^	0.00	20 and 26–29
12	N˙ + O_3_ → NO + O_2_	6.00 × 10^07^	0.00	12 and 14
13	N_2_ + O_3_ → N_2_O + O_2_	6.00 × 10^07^	90.00	30
14	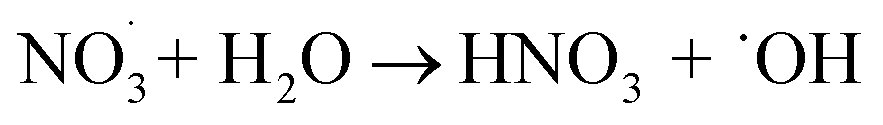	2.99 × 10^−02^	52.46	18
15	N_2_O_3_ + H_2_O → 2HNO_2_	1.95 × 10^12^	71.86	13 and 31
16	N_2_O_4_ + H_2_O → HNO_2_ + HNO_3_	6.30 × 10^14^	5.00	13 and 31
17	NO + NO_2_ → N_2_O_3_	1.41 × 10^09^	5.00	13,15 and 20
18	2NO_2_ → N_2_O_4_	2.52 × 10^11^	8.00	13 and 15
19	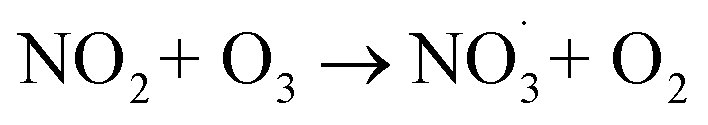	2.87 × 10^11^	6.11	6 and 13–15
20	N_2_O_5_ + H_2_O → 2HNO_3_	1.51 × 10^02^	0.00	13–15
21	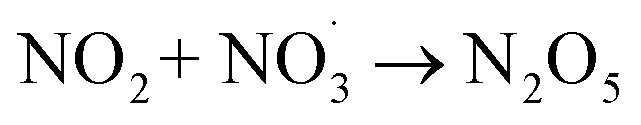	1.58 × 10^14^	8.33	6 and 12–15
22	NO_2_ + ˙OH → HNO_3_	6.90 × 10^17^	0.00	12 and 14
23	H_2_O_2_ → 2˙OH	1.30 × 10^17^	45.50	12
24	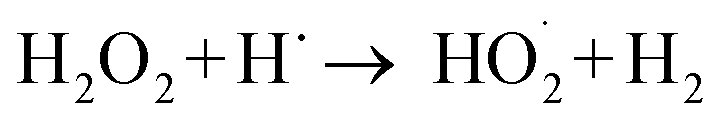	1.60 × 10^12^	3.80	12 and^14^
25	O_3_ + H_2_O → O_2_ + H_2_O_2_	6.62 × 10^01^	0.00	12 and 14
26	H_2_O → ˙OH + H˙	6.62 × 10^01^	0.00	12 and 14
27	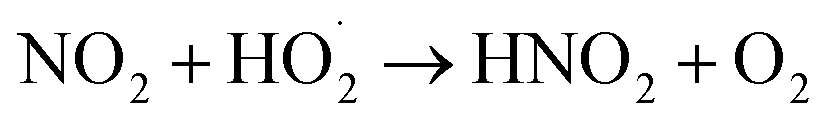	2.20 × 10^−01^	0.00	12 and 14
28	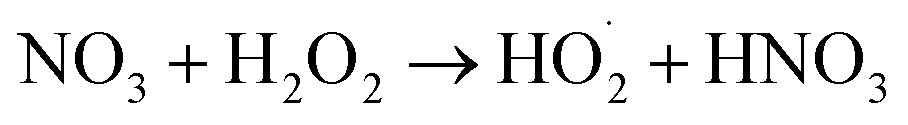	1.21 × 10^09^	0.00	14
29	N_2_O_3_ → NO + NO_2_	5.78 × 10^11^	270.03	13 and 31
30	N_2_O_4_ → 2NO_2_	3.23 × 10^08^	12.18	13 and 31
31	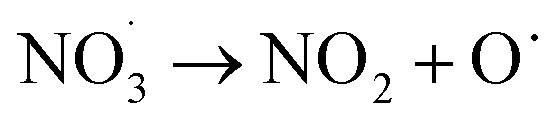	1.13 × 10^11^	40.92	18
32	SO_2_ + O_3_ → SO_3_ + O_2_	6.80 × 10^09^	6.90	32
33	SO_3_ + O˙ → SO_2_ + O_2_	9.00 × 10^12^	1.00	33

## 3. Validation of the reaction mechanism

In this work, the mechanism constructed using the MRN method is validated by comparing the evolution of species concentrations with experimental data and simulated results reported in other publications.^6,10,12–15^ All mechanism simulations were carried out using the Plug Flow Reactor (PFR) model embedded in the CHEMKIN software.

### 3.1 Validation of the O_3_ self-decomposition reaction mechanism

First, the O_3_ self-decomposition mechanism consisting of 5 species (O_3_, O_2_, O, Ar, and He) and 5 reactions is constructed. Numerical simulations of O_3_ self-decomposition are conducted under various working conditions, and the simulated results are compared with the corresponding reference data, as shown in Fig. 2. In a previous study, Wang *et al.* showed the remaining percentage of O_3_ with time at 273–523 K, starting from an initial O_3_ concentration of 4400 ppm.^10^ The results of this study were taken as the reference data, as shown in Fig. 2. The comparative results demonstrate that the numerical predictions of the reaction mechanism in this study agree well with the O_3_ self-decomposition experimental data, confirming the reliability and accuracy of the mechanism for O_3_ self-decomposition prediction under various working conditions.

**Fig. 2:**
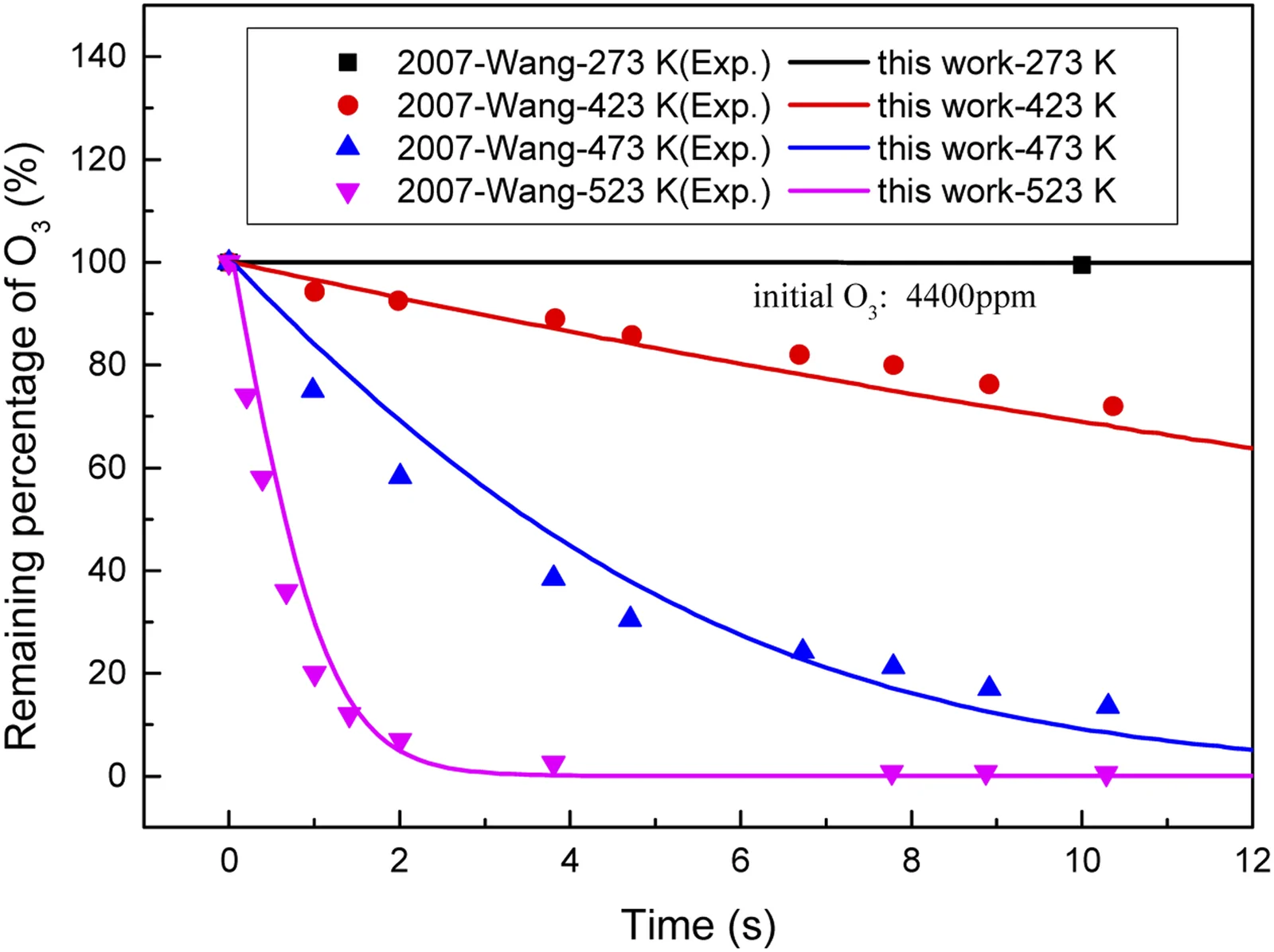
Simulation results of O_3_ self-decomposition under different working conditions and the corresponding reference data.

### 3.2 Validation of the O_3_ oxidation denitrification reaction mechanism

On the basis of the established O_3_ self-decomposition mechanism, 17 nitrogen-containing species and 26 nitrogen-related reactions are supplemented to construct the O_3_ oxidation denitrification mechanism. Fig. 3 compares the numerical simulation results and the corresponding reference values of NO, NO_2_, N_2_O_3_, N_2_O_4_, N_2_O_5_ and O_3_ under various working conditions to verify the denitrification accuracy of the proposed mechanism. Fig. 3(a) presents the variations in the molar fractions of NO and NO_2_ with the corresponding reference data under different molar ratios of O_3_ to NO at 443 K. Fig. 3(b) shows the comparative molar fraction results of NO under different O_3_ : NO molar ratios and temperatures. Fig. 3(c) displays the molar fraction comparison of N_2_O_5_ under various O_3_ : NO molar ratios and temperature conditions. Fig. 3(d) illustrates the molar fraction variations of NO, NO_2_ and O_3_ with different O_3_ : NO molar ratios at 353 K. Fig. 3(e) compares the molar fractions of N_2_O_3_ and N_2_O_5_ at diverse O_3_ : NO molar ratios. Fig. 3(f) shows the molar fraction changes in N_2_O_4_ at different temperatures. The comprehensive comparison of the six groups of results indicates that the numerical predictions of O, NO_2_, N_2_O_3_, N_2_O_4_, N_2_O_5_ and O_3_ based on the proposed mechanism are basically consistent with the experimental data under various working conditions. Moreover, the developed mechanism exhibits comparable or even better performance than other reported mechanisms.^6,12–15^ Therefore, the established mechanism can effectively describe the conversion characteristics of various NO_*x*_ under different working conditions and ensure the accuracy of O_3_ oxidation denitrification predictions under diverse initial conditions.

**Fig. 3:**
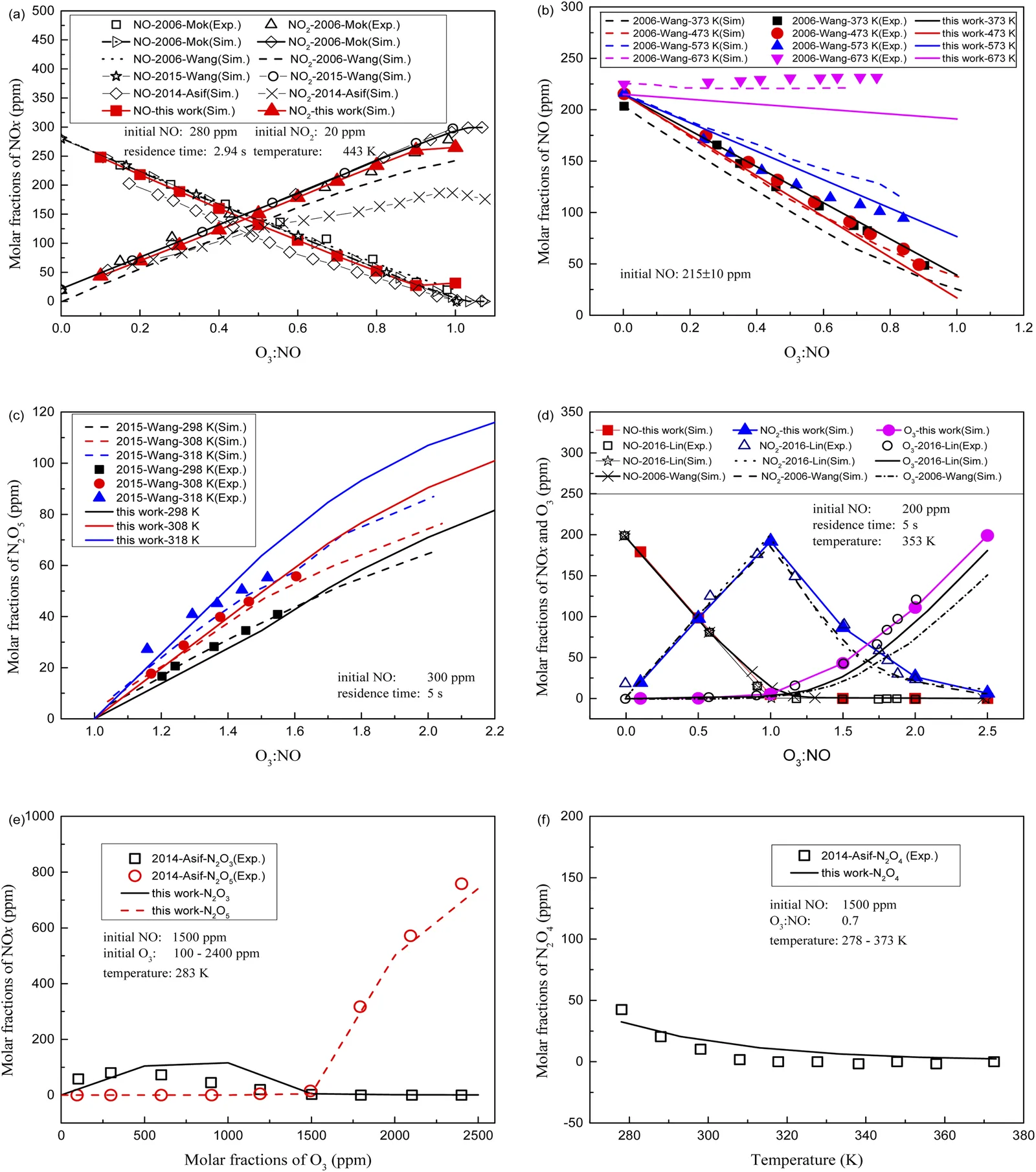
Molar fractions of O_3_ and NO_*x*_ under different conditions. (a) Molar fractions of NO and NO_2_ at different O_3_ : NO ratios. (b) Molar fractions of NO at different temperatures and O_3_ : NO ratios. (c) Molar fractions of N_2_O_5_ at different temperatures and O_3_ : NO ratios. (d) Molar fractions of NO, NO_2_, and O_3_ at different O_3_ : NO ratios. (e) Molar fractions of N_2_O_3_ and N_2_O_5_ at different O_3_ : NO ratios. (f) Molar fractions of N_2_O_4_ at different temperatures.

### 3.3 Validation of the O_3_ oxidation desulfurization reaction mechanism

Based on the established O_3_ self-decomposition mechanism, two sulfur-containing species and two sulfur-related reactions are added to develop the O_3_ oxidation desulfurization mechanism. The accuracy of the developed O_3_ oxidation desulfurization and denitrification mechanism is validated using the experimental data reported by Wang *et al.*, as shown in Fig. 4.^10^ The figure presents the oxidation efficiency of SO_2_ at different temperatures. The comparison shows that the numerical predictions of the minimal reaction mechanism established in this work are in good agreement with the experimental data. A slight discrepancy of 6.90% is observed at 573 K, which is acceptable, thereby ensuring the prediction accuracy of the mechanism for O_3_ oxidation desulfurization under various initial conditions. Furthermore, the incorporation of nitrogen-related species and reactions into the desulfurization mechanism barely affects the simulation results. In addition, previous studies have demonstrated that the presence of SO_2_ has a negligible effect on the O_3_-induced oxidation of NO_*x*_.^10^ Accordingly, the two sulfur-containing species and two sulfur-related reactions can be directly integrated into the O_3_ oxidation denitrification minimal reaction mechanism to form a complete O_3_ oxidation desulfurization and denitrification mechanism.

**Fig. 4:**
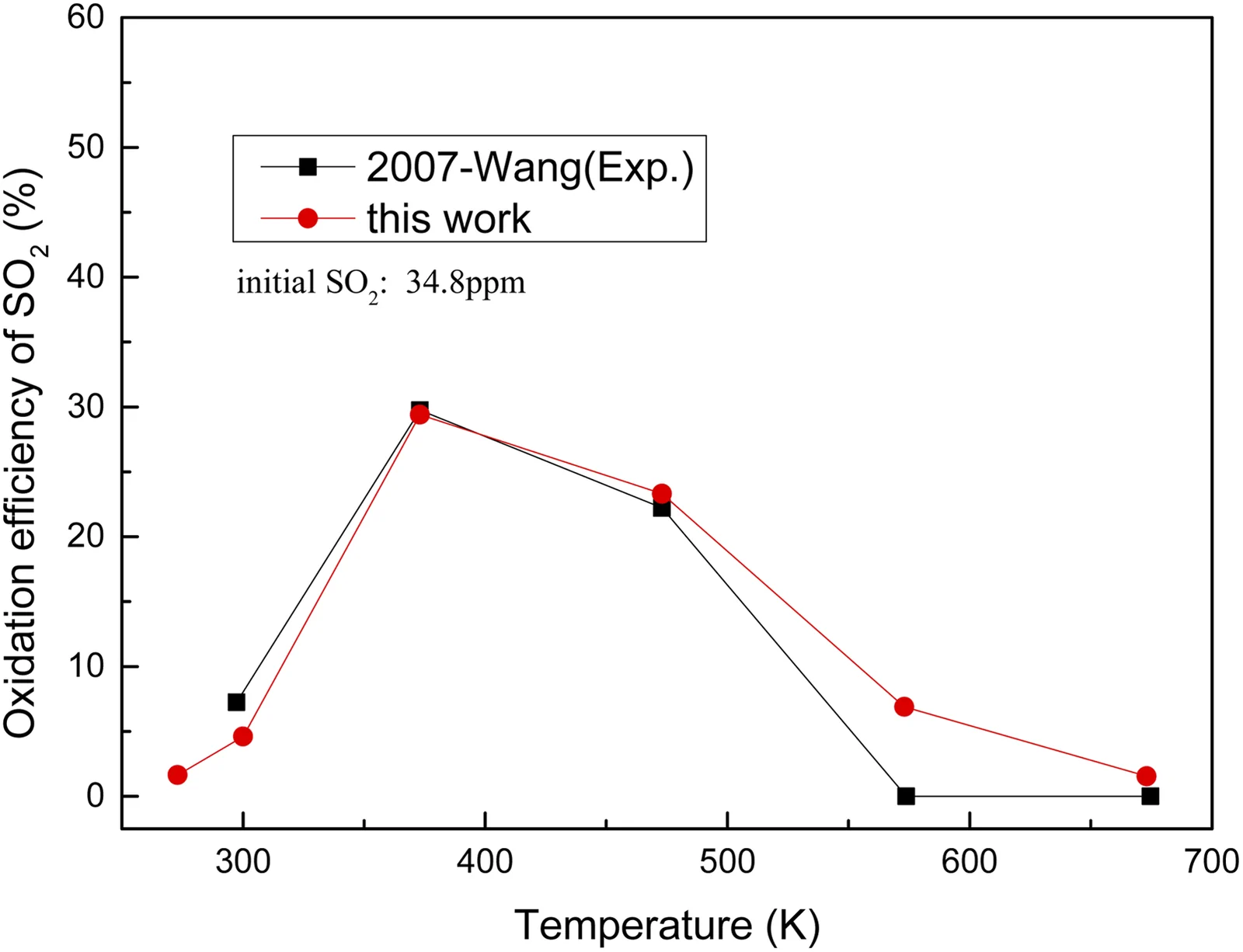
Oxidation efficiency of SO_2_ at different temperatures.

The above results verify the accuracy of the simulation for O_3_ self-decomposition, O_3_ oxidation denitrification and O_3_ oxidation desulfurization under different initial conditions, which guarantees the reliability of the reaction mechanism in predicting the desulfurization and denitrification performance. In addition, the mechanism proposed in this study contains a more comprehensive set of species than those in other related studies. Meanwhile, it features both high accuracy and concise pathway. Hence, it is well suited for engineering calculations.

## 4. Conclusions

Based on the MRN method, the O_3_ oxidation desulfurization and denitrification mechanism is established in this study. The reaction mechanism contains 33 reactions and 24 species, covering a more complete set of species. Meanwhile, it balances accuracy and simplicity, making it more suitable for engineering calculations. In addition, comparing the experimental and simulation data from previous studies verifies the numerical results of the O_3_ self-decomposition at 273–523 K, O_3_ oxidation denitrification at 278–673 K and O_3_ oxidation desulfurization at 273–673 K. Thus, the accuracy of the proposed mechanism is confirmed under different working conditions.

## Conflicts of interest

There are no conflicts to declare.

## Data Availability

The authors confirm that the data and materials supporting the findings of this study are available within the article. More research data and materials are available from the corresponding author upon reasonable request.
